# Effect Analysis of Mineral Salt Concentrations on Nosiheptide Production by *Streptomyces actuosus* Z-10 Using Response Surface Methodology

**DOI:** 10.3390/molecules191015507

**Published:** 2014-09-26

**Authors:** Wei Zhou, Xiaohui Liu, Pei Zhang, Pei Zhou, Xunlong Shi

**Affiliations:** 1 Department of Chemistry, Fudan University, 220 Handan Road, Shanghai 200433, China; 2 College of Chemistry and Chemical Engineering, Shanghai University of Engineering Science, 333 Longteng Road, Shanghai 201620, China; 3 CAS Key Laboratory of Synthetic Chemistry of Natural Substances, Shanghai Institute of Organic Chemistry, Chinese Academy of Sciences, 345 Lingling Road, Shanghai 200032, China; 4 School of Pharmacy, Fudan University, 826 Zhangheng Road, Shanghai 201203, China

**Keywords:** nosiheptide, *Streptomyces actuosus*, optimization, Plackett-Burman design, response surface methodology

## Abstract

The objective of this study was to develop an optimal combination of mineral salts in the fermentation medium for nosiheptide (Nsh) production using statistical methodologies. A Plackett-Burman design (PBD) was used to evaluate the impacts of eight mineral salts on Nsh production. The results showed that among the no-significant factors, CaCO_3_, and K_2_HPO_4_·3H_2_O had positive effects, whereas FeSO_4_·7H_2_O, CuSO_4_·5H_2_O, and ZnSO_4_·7H_2_O had negative effects on Nsh production. The other three significant factors (Na_2_SO_4_, MnSO_4_·H_2_O, and MgSO_4_·7H_2_O) were further optimized by using a five-level three-factor central composite design (CCD). Experimental data were fitted to a quadratic polynomial model, which provided an effective way to determine the interactive effect of metal salts on Nsh production. The optimal values were determined to be 2.63, 0.21, and 3.37 g/L, respectively. The model also ensured a good fitting of scale-up Nsh batch fermentation with a maximum production of 1501 mg/L, representing a 1.56-fold increase compared to the original standard condition. All these results revealed that statistical optimization methodology had the potential to achieve comprehensive optimization in Nsh fermentation behaviors, which indicates a possibility to establish economical large-scale production of Nsh.

## 1. Introduction

Secondary metabolites of microorganisms can serve as good sources of drugs, foods, nutrients, and chemical reagents. Nosiheptide (Nsh) isolated from *Streptomyces actuosus* is a thiopeptide antibiotic belonging to the thiazole heterocyclic peptide antibiotic family [1]. It acts on the 50S ribosomal subunit by tightly binding to the complex of 23S rRNA with ribosomal protein L-11 and inhibiting GTP hydrolysis activities by elongation factors [2]. It has been widely used in veterinary medicine due to its effectiveness against Gram-positive bacteria, and as a commercial feed additive to increase weight gain in poultry and pigs without leaving any residues in the body [3].

There is a compelling need to develop new strategies and antibiotics with a novel mechanism of action because of the increasing resistance of pathogenic bacteria to existing antibiotics [4]. Thiopeptide antibiotic scaffolds typically present with several dehydrated amino acids and multiple thiazole or oxazole rings that are highly active against clinically relevant methicillin-resistant *Staphylococcus aureus* (MRSA), methicillin-resistant *Enterococcus faecium* (MREF), penicillin-resistant *Streptococcus pneumoniae* (PRSP), and vancomycin-resistant enterococci (VRE). For these reasons, research interest in this field has been on the rise over the past years [5,6,7]. Nsh is distinguished from other thiopeptide natural products by an indolic acid macrothiolactone group which forms the smaller B ring and a peculiar 3-hydroxypyridine group in the larger A ring [8]. The structure and stereochemistry of Nsh have been elucidated by X-ray crystallography and NMR spectroscopic methods [1]. However, to the best of our knowledge, total synthesis of Nsh has not yet been reported till date, and fermentation of *S. actuosus* remains the only way for Nsh production, an approach that has been the subject of considerable criticism due to high cost and low yield [9,10,11].

Medium optimization by the classical one-factor-at-a-time method is laborious and time-consuming, especially when the number of variables is large. A more efficient alternative in microbial system is to use statistical methods. Plackett-Burman design (PBD) is an efficient tool to screen for important factors among a large number of variables using a limited number of experiments, and allows estimation of random error variability and test of the statistical significance of parameters [12]. Response surface methodology (RSM) is an effective statistical method that uses quantitative data from appropriate experiments to evaluate multiple parameters and their interactions, determine the optimum conditions of factors for desirable responses, and evaluate the relative significance of affecting factors even in the presence of complex interactions [13,14]. These methods have already been widely and successfully applied to modeling of fermentation processes [15,16,17], but their application to Nsh production has not been reported in the literature.

The culture medium for antibiotic-producing microorganisms usually contains varying amounts of mineral elements necessary for the production of antibiotics. The regulatory effect of medium mineral content on microbial secondary metabolism has been observed in a variety of species [18], but their effect on Nsh production remains largely unknown. In this study, mineral salt factors in the original fermentation medium of Nsh were selected and the critical salts were identified by PBD and then further optimized through a properly designed CCD. The model of Nsh fermentation process was presented by RSM for the first time, so as to determine the best set of salts combination. In addition, scale-up fermentation process of *S. actuosus* under optimized conditions was also investigated. It is hoped that the results of this study could be helpful for improving industrial large-scale production of Nsh.

## 2. Results and Discussion

### 2.1. Determination of Critical Salts by PBD

The effects of eight mineral salts, CaCO_3_ (X_1_), Na_2_SO_4_ (X_2_), K_2_HPO_4_·3H_2_O (X_3_), MnSO_4_·H_2_O (X_4_), MgSO_4_·7H_2_O (X_5_), FeSO_4_·7H_2_O (X_6_), CuSO_4_·5H_2_O (X_7_), and ZnSO_4_·7H_2_O (X_8_), on Nsh production were investigated by PBD. Table 1 shows that Nsh production varies significantly from 801 to 1341 mg/L throughout the twelve trials, thus highlighting the need to optimize these mineral salts to maximize Nsh production (Table 2). X_2_ (Na_2_SO_4_; *p* = 0.012), X_4_ (MnSO_4_·H_2_O; *p* = 0.013), and X_5_ (MgSO_4_·7H_2_O; *p* = 0.031) are identified to have significant effects on Nsh production. The *F* and *p* values based on Fisher’s test for ANOVA are 10.0 and 0.042, respectively, suggesting that the model is statistically significant at a 95% significance level. In addition, *R^2^* (96.38%) and Adj*R^2^* (86.74%) are also very high to advocate for a high significance of the model.

**Table 1:** Plackett-Burman experimental design and Nsh production.

Runs	Real Levels (Coded Levels)								Nsh Production (mg/L)
	CaCO_3_ (g/L)	Na_2_SO_4_(g/L)	K_2_HPO_4_·3H_2_O (g/L)	MnSO_4_·H_2_O (g/L)	MgSO_4_·7H_2_O (g/L)	FeSO_4_·7H_2_O (g/L)	CuSO_4_·5H_2_O (g/L)	ZnSO_4_·7H_2_O (g/L)	
1	7.5 (+)	1.0 (−)	0.5 (+)	0.1 (−)	1.0 (−)	0.01 (−)	0.03 (+)	0.09 (+)	840.3 ± 21.3
2	7.5 (+)	5.0 (+)	0.1 (−)	0.5 (+)	1.0 (−)	0.01 (−)	0.01 (+)	0.09 (+)	1258.6 ± 30.6
3	2.5 (−)	5.0 (+)	0.5 (+)	0.1 (−)	5.0 (+)	0.01 (−)	0.01 (−)	0.03 (−)	1121.5 ± 33.6
4	7.5 (+)	1.0 (−)	0.5 (+)	0.5 (+)	1.0 (−)	0.05 (+)	0.01 (−)	0.03 (−)	940.3 ± 26.2
5	7.5 (+)	5.0 (+)	0.1 (−)	0.5 (+)	5.0 (+)	0.01 (−)	0.03 (+)	0.03 (−)	1341.2 ± 31.5
6	7.5 (+)	5.0 (+)	0.5 (+)	0.1 (−)	5.0 (+)	0.05 (+)	0.01 (−)	0.09 (+)	1128.5 ± 27.9
7	2.5 (−)	5.0 (+)	0.5 (+)	0.5 (+)	1.0 (−)	0.05 (+)	0.03 (+)	0.03 (−)	1160.4 ± 22.3
8	2.5 (−)	1.0 (−)	0.5 (+)	0.5 (+)	5.0 (+)	0.01 (−)	0.03 (+)	0.09 (+)	1100.9 ± 25.4
9	2.5 (−)	1.0 (−)	0.1 (−)	0.5 (+)	5.0 (+)	0.05 (+)	0.01 (−)	0.09 (+)	1029.6 ± 31.5
10	7.5 (+)	1.0 (−)	0.1 (−)	0.1 (−)	5.0 (+)	0.05 (+)	0.03 (+)	0.03 (−)	951.3 ± 29.7
11	2.5 (−)	5.0 (+)	0.1 (−)	0.1 (−)	1.0 (−)	0.05 (+)	0.03 (+)	0.09 (+)	833.7 ± 23.6
12	2.5 (−)	1.0 (−)	0.1 (−)	0.1 (−)	1.0 (−)	0.01 (−)	0.01 (−)	0.03 (−)	801.7 ± 19.8

Among the non-significant factors, CaCO_3_ (X_1_) and K_2_HPO_4_·3H_2_O (X_3_) have positive effects, whereas FeSO_4_·7H_2_O (X_6_), CuSO_4_·5H_2_O (X_7_), and ZnSO_4_·7H_2_O (X_8_) have negative effects on Nsh production. Thus, high levels (+) of X_1_ (7.5 g/L CaCO_3_) and X_3_ (0.5 g/L K_2_HPO_4_·3H_2_O), and low levels (−) of X_6_(0.01 g/L FeSO_4_·7H_2_O), X_7_(0.01 g/L CuSO_4_·5H_2_O), and X_8_(0.03 g/L ZnSO_4_·7H_2_O) are selected as a fixed composition used in following experiments.

**Table 2:** Statistical analysis of Plackett-Burman design.

Variables ^#^	Effect	Coefficient	SE-Coefficient	T-Value	*p*-Value
Constant		1042.33	18.16	57.41	0.000
X_1_	68.73	34.37	18.16	1.89	0.155
X_2_	196.63	98.32	18.16	5.42	0.012 *
X_3_	12.63	6.32	18.16	0.35	0.751
X_4_	192.33	96.17	18.16	5.30	0.013 *
X_5_	139.67	69.83	18.16	3.85	0.031 *
X_6_	−70.07	−35.03	18.16	−1.93	0.149
X_7_	−8.73	−4.37	18.16	−0.24	0.825
X_8_	−20.80	−10.40	18.16	−0.57	0.607
*R^2^*	96.38%				
Adj *R^2^*	86.74%				

^#^ X_1_, CaCO_3_; X_2_, Na_2_SO_4_; X_3_, K_2_HPO_4_·3H_2_O; X_4_, MnSO_4_·H_2_O; X_5_, MgSO_4_·7H_2_O; X_6_, FeSO_4_·7H_2_O; X_7_, CuSO_4_·5H_2_O; X_8_, ZnSO_4_·7H_2_O. * *p* < 0.05.

### 2.2. Statistical Optimization with CCD

**Figure 1:**
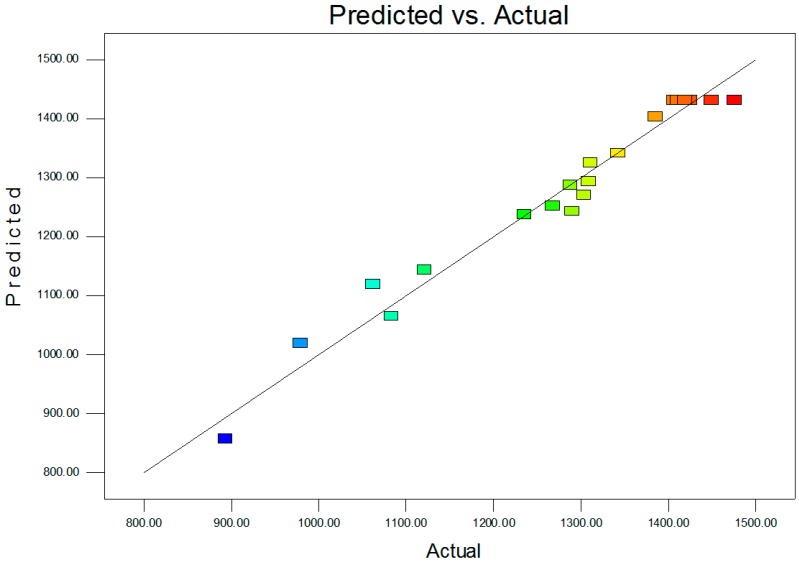
Predicted *versus* actual values of the response function.

CCD was employed to investigate the interactive effects of the three significant factors (X_2_ (Na_2_SO_4_), X_4_ (MnSO_4_·H_2_O), and X_5_ (MgSO_4_·7H_2_O)) on Nsh production (Table 3). Twenty experimental runs including six center points were conducted with the three independent variables at five levels (−1.732, −1, 0, 1, 1.732). In our study, the ANOVA results of the quadratic regression model indicate that Equation 1 results in a highly significant model, as evidenced by the *F* value (36.85) and *p* value (<0.0001) in Table 4. The linear term coefficients of X_2_, X_4_, and X_5_, the quadric term coefficients of X_2_^2^, X_4_^2^, and X_5_^2^, and the cross-product coefficients of X_2_X_4_ and X_2_X_5_ have significant effects on Nsh production. However, the interaction coefficient of X_4_X_5_ seems to have no significant effect on Nsh production. The high value of *R^2^* (0.9707) indicates a good fit of the quadratic regression model to the observed responses [19]. The value of Adj*R^2^* is also very high (0.9444), further demonstrating a high significance of the model, and the predicted *R^2^* value of 0.8176 for Nsh production is in reasonable agreement with the Adj*R^2^*. All these results indicate that the response equation provides a suitable and reasonable model for CCD experiments. The model also shows a large Adeq Precision value (20.95), so it is supposed to be adequate for prediction within the range of variables employed [20]. In addition, the relatively low coefficient of variation (3.04) indicates good precision and reliability of the experiments (Figure 1). The application of multiple regression analysis to the experimental data results in the following second-order polynomial equation to determine the production of Nsh:

Y = −26.73 + 428.21X_2_ + 3730.44X_4_ + 317.66X_5_ − 231.83X_2_X_4_ − 43.52X_2_X_5_ − 164.73X_4_X_5_ − 44.24X_2_^2^ − 6177.21X_4_^2^ − 25.10X_5_^2^ (1)

**Table 3:** Central composite experiment design with the three significant variables and Nsh production.

Runs	Real Levels (Coded Levels)			Nsh Production (mg/L)	
	Na_2_SO_4_(g/L)	MnSO_4_·H_2_O (g/L)	MgSO_4_·7H_2_O (g/L)	Actual	Predicted
1	1.05 (−1)	0.08 (−1)	0.84 (−1)	892.4 ± 25.8	857.6
2	3.94 (1)	0.08 (−1)	0.84 (−1)	1303.5 ± 37.7	1271.5
3	1.05 (−1)	0.31 (1)	0.84 (−1)	1083.2 ± 41.9	1065.7
4	3.94 (1)	0.31 (1)	0.84 (−1)	1310.8 ± 40.9	1325.5
5	1.05 (−1)	0.08 (−1)	3.15 (1)	1290.3 ± 33.4	1243.5
6	3.94 (1)	0.08 (−1)	3.15 (1)	1308.5 ± 37.6	1293.9
7	1.05 (−1)	0.31 (1)	3.15 (1)	1342.2 ± 35.7	1342.2
8	3.94 (1)	0.31 (1)	3.15 (1)	1235.7 ± 29.8	1238.4
9	0 (−1.732)	0.2 (0)	2.0 (0)	978.3 ± 26.8	1019.8
10	5.0 (1.732)	0.2 (0)	2.0 (0)	1287.2 ± 31.4	1288.4
11	2.5 (0)	0 (−1.732)	2.0 (0)	1061.7 ± 22.4	1120.1
12	2.5 (0)	0.4 (1.732)	2.0 (0)	1267.9 ± 39.8	1252.3
13	2.5 (0)	0.2 (0)	0 (−1.732)	1120.1 ± 27.3	1144.6
14	2.5 (0)	0.2 (0)	4.0 (1.732)	1385.2 ± 30.8	1403.4
15	2.5 (0)	0.2 (0)	2.0 (0)	1475.9 ± 30.5	1431.3
16	2.5 (0)	0.2 (0)	2.0 (0)	1406.2 ± 33.4	1431.3
17	2.5 (0)	0.2 (0)	2.0 (0)	1411.1 ± 28.4	1431.3
18	2.5 (0)	0.2 (0)	2.0 (0)	1449.2 ± 33.1	1431.3
19	2.5 (0)	0.2 (0)	2.0 (0)	1425.7 ± 25.3	1431.3
20	2.5 (0)	0.2 (0)	2.0 (0)	1419.5 ± 28.3	1431.3

**Table 4:** Analysis of variance (ANOVA) for CCD.

Term ^#^	DF	Sum of Squares	*F*-Value	*p*-Value
Model	9	4.975 × 10^5^	36.85	<0.0001 **
X_2_	1	84154.24	56.11	<0.0001 **
X_4_	1	20394.91	13.60	0.0042 **
X_5_	1	78146.16	52.10	<0.0001 **
X_2_X_4_	1	11873.40	7.92	0.0184 *
X_2_X_5_	1	66066.12	44.05	<0.0001 **
X_4_X_5_	1	5995.12	4.00	0.0735
X_2_^2^	1	1.344 × 10^5^	89.61	<0.0001 **
X_4_^2^	1	1.051 × 10^5^	70.08	<0.0001 **
X_5_^2^	1	43262.75	28.84	0.0003 **
Residual	10	14999.35		
lack-of-fit	5	11481.14	3.26	0.1101
Pure error	5	3518.21		
Corrected Total	19	5.125 × 10^5^		
*R^2^*	0.9707		Pred*R^2^*	0.8176
Adj*R^2^*	0.9444		Adeq Precision	20.948

^#^ X_2_, Na_2_SO_4_; X_4_, MnSO_4_·H_2_O; X_5_, MgSO_4_·7H_2_O. * Significant at *p <* 0.05. ** High significant at *p <* 0.01.

**Figure 2:**
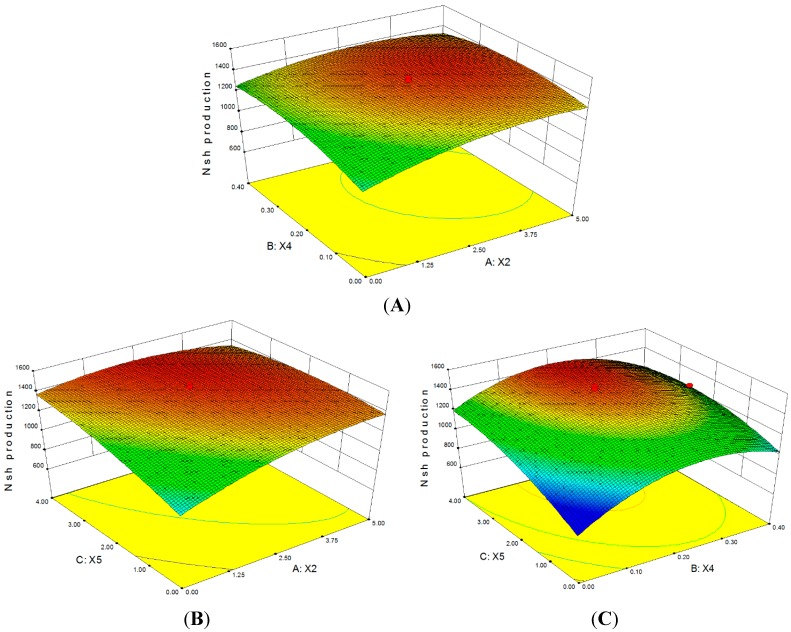
Three-dimensional (3D) response surface plots for dependent variables as a function of (**A**) Na_2_SO_4_ (X_2_) and MnSO_4_·H_2_O (X_4_); (**B**) Na_2_SO_4_ (X_2_) and MgSO_4_·7H_2_O (X_5_); and (**C**) MnSO_4_·H_2_O (X_4_) and MgSO_4_·7H_2_O (X_5_).

The relationships between the variables and responses could be better illustrated by the 3D plots obtained from the predicted model using Design-Expert software (Design Expert 8.0.7.1, Stat-Ease Inc., Minneapolis, MN, USA). These plots were generated for the pairwise combination of the three variables while keeping the third one at “0” level. Figure 2A shows the interaction between Na_2_SO_4_ (X_2_) and MnSO_4_·H_2_O (X_4_) while keeping MgSO_4_·7H_2_O (X_5_) at zero level (2.0 g/L). The results demonstrate that with the increase of Na_2_SO_4_ and MnSO_4_·H_2_O to 3.09 g/L and 0.21 g/L, respectively, Nsh production increases to a maximum of 1449 mg/L and then decreases thereafter. Figure 2B shows the interactive effect of Na_2_SO_4_ and MgSO_4_·7H_2_O while keeping MnSO_4_·H_2_O at 0.2 g/L. Nsh production increases to the optimum level of 1458 mg/L with the increase of Na_2_SO_4_ and MgSO_4_·7H_2_O to 2.75 g/L and 3.44 g/L, respectively. Figure 2C shows the interactive effect of MnSO_4_·H_2_O and MgSO_4_·7H_2_O while keeping Na_2_SO_4_ at 2.5 g/L. The maximum Nsh production of 1459 mg/L is obtained when 0.21 g/L MnSO_4_·H_2_O and 3.43 g/L MgSO_4_·7H_2_O are used. By taking the first-order partial derivative of Equation (1), the optimum concentrations of Na_2_SO_4_, MnSO_4_·H_2_O, and MgSO_4_·7H_2_O are determined to be 2.63, 0.21, and 3.37 g/L, respectively, and the resulting model predicts a maximum Nsh production of 1459.55 mg/L.

### 2.3. Verification of Optimal Conditions in Scale-up Fermentation

**Figure 3:**
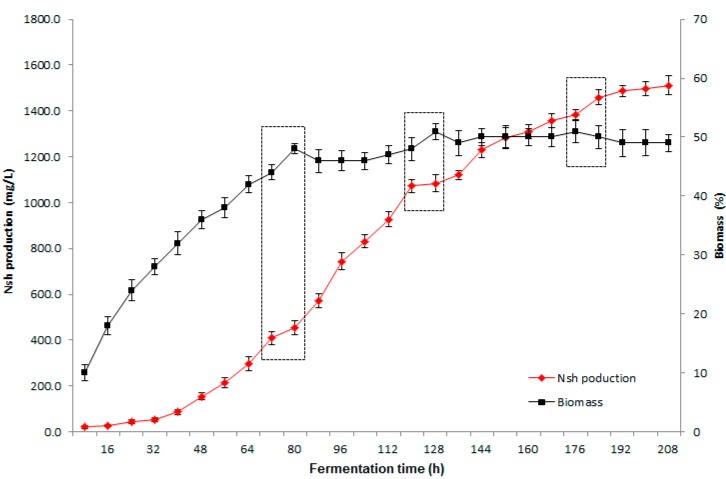
Time courses of biomass (■) and Nsh production (♦) under the optimal medium composition. The dashed frames indicate the time points of feeding.

Figure 3 shows the time courses of Nsh production and biomass in 100 L fermentor using the optimal medium. It is noted that Nsh biomass increases rapidly at first until a stationary phase is reached at about 80 h. The addition of sterilized feeding solution at 72 h, 120 h and 168 h influences the biomass increase and Nsh production (see the dashed frame in Figure 3), probably due to that the quick increase of carbon source improves the primary metabolic process, which consumes more ATP and represses related biosynthetic enzymes, and thus as a result inhibits the secondary metabolite production [21]. Nsh production increases rapidly from 48 to 120 h, and then increases slightly until a maximum of 1501 mg/L is reached at the end of fermentation, which is 3% higher than the calculated value from the suggested model, indicating high accuracy and generalization ability.

Microbial fermentation remains the major method for the production of antibiotics, and bioprocess optimization is one of the most efficient ways to improve the yield [22,23]. However, it is very inconvenient to optimize such a complex bioprocess by the one-factor-at-a-time approach, especially for Nsh fermentation because of the long-time lasting process and complex medium composition [24]. Thus, statistical optimization and modeling of the fermentation process are urgently required. Recently, Elman neural network model [11] and hybrid model [25] have been successfully applied to Nsh batch fermentation, and proven to be precise and useful for process optimization. In this study, RSM has been applied to the optimization and modeling of Nsh fermentation for the first time. Although only mineral slats are investigated, Nsh production is greatly improved and a good predictive model is obtained, thus RSM is effective in optimizing Nsh bioprocess.

Our previous studies have demonstrated the importance of metal salts, Na_2_SO_4_and MnSO_4_, for the production of Nsh by *S. actuosus* [26,27]. However, there is also a need to investigate the effects of other metal ions, such as Ca^2+^, Mg^2+^, Zn^2+^, Cu^2+^ and Fe^2+^, as these metal ions have also been reported to significantly affect the metabolite production by different microorganisms. For example, Ca^2+^ has been identified as an inhibitory substance for the biosynthesis of efrotomycin in resting cells of *Nocradia lactamdurans* [28]. Mg^2+^ plays a catalytic role as the cofactor of the key enzymes involved in the glucose metabolite to stimulates DNA and protein synthesis [29]. Mn^2+^ enriches the biotechmycin precursor pool such as propionic acid and thus improves the yield of biotechmycin by *Streptomyces spiramyceticus* [30]. Fe^2+^ improves the binding affinity of UDP-pyrophophorylase towards glucose-1-phosphate and Cu^2+^ contributes towards the interaction between UDP-glucose dehydrogenase and UDP-glucose to increase the production of hyaluronic acid by *Lactobacillus acidophilus* FTDC 1231 [31]. In this experiment, Na_2_SO_4_ and MnSO_4_ significantly increase Nsh production in RSM statistical methodologies, which is consistent with the results obtained in our previous single-factor experiments. MgSO_4_ is essential for the production of Nsh, probably due to its effect on the Mg-requiring enzymes involved in the synthesis of peptide antibiotics. Recent years have seen considerable progress in the biosynthesis of thiopeptide antibiotics, and more and more enzymes participating in Nsh biosynthesis have been characterized from gene cluster [32,33,34]. The impact of metal ions acting as cofactors for a large number of enzymes and iron-containing proteins involved in Nsh metabolism needs to be further studied in order to improve Nsh production with economically more viable mineral salts.

Thiopeptide antibiotics have emerged as a promising class of natural products that have not been used for human therapy. However, they are constantly questioned as lead molecules to combat infectious diseases. The bismacrocyclic Nsh stands out among them as having the highest potency against multidrug-resistant strains. This study emphasizes the importance of optimizing culture variables using RSM to maximize Nsh production, as Nsh production under optimal conditions is increased by 1.56 times (1501 mg/L) as compared to that under non-optimal conditions. The highest yield was reported to be 1746 mg/L from S-adenosylmethionine synthetase (SAM) over-expressed recombinant mutant in our previous study [35]. Due to the critical requirement of strain stability in industrial production, we preferred to choose more stable *S. actuosus* Z-10 to research the statistical optimization and scale up fermentation in this study, and the results from this statistical optimization method may indicate further application in the SAM recombinant strain to archive much higher yield. Overall, our findings suggest the utility of composted salts for the economical production of a higher-value product and further intendance to verify Nsh fermentation behaviors for achieving comprehensive optimization using the response surface methodology.

## 3. Experimental Section

### 3.1. Microorganisms and Chemicals

*S. actuosus* Z-10 was obtained from American Type Culture Collection (ATCC 25421) and mutated by ultraviolet radiation in our lab [26]. Stock cultures were routinely maintained on 2% agar slant composed of 40 g/L glucose, 10 g/L peptone, 1 g/L (NH_4_)_2_SO_4_ and 5 g/L CaCO_3_ with a pH of 7.0–7.2, subcultured at regular intervals of one month, and stored at −80 °C for long-term preservation.

Nsh standard was kindly supplied by Prof. Floss of the Department of Chemistry, University of Washington. HPLC-grade acetonitrile was purchased from Fisher Chemicals (Fair Lawn, NJ, USA). All other chemicals were of analytical grade and obtained from Sinopharm Chemical Reagent Co., Ltd. (Shanghai, China).

### 3.2. Fermentation Conditions

The seed medium was composed of 10 g/L corn steep liquor, 20 g/L starch, 20 g/L sucrose, 5 g/L Na_2_SO_4_, 10 g/L NaCl, and 5 g/L CaCO_3_. The seed culture was prepared in 250 mL sterile flask containing 50 mL of seed medium, and incubated at 220 revolutions per minute (rpm) at 28 °C for 3 days. The original fermentation medium was composed of 40 g/L glucose, 40 g/L starch, 30 g/L soy meal, 2 g/L (NH_4_)_2_SO_4_, 2.5 g/L NaCl, 5 g/L CaCO_3_, 2 g/L Na_2_SO_4_, 0.4 g/L K_2_HPO_4_·3H_2_O, 0.1 g/L MnSO_4_·H_2_O, 1 g/L MgSO_4_·7H_2_O, 0.03 g/L FeSO_4_·7H_2_O, 0.01 g/L CuSO_4_·5H_2_O, and 0.05 g/L ZnSO_4_·7H_2_O. The media pH was about 7.0 before sterilization and was not adjusted any further. The 250 mL flasks with 30 mL of medium were sterilized at 121 °C for 30 min, and then inoculated with 10% (v/v) of the above seed culture at 220 rpm at 28 °C for 9 days. A sterilized solution of 40% glucose and 2.5% (NH_4_)_2_SO_4_ (w/v) in water was fed at 72 h (2 mL), 120 h (1 mL) and 168 h (1 mL) to supply carbon and nitrogen sources for cell growth. The Nsh production under the original conditions was 959 mg/L.

### 3.3. Plackett-Burman Design

The eight mineral salts in the original medium were chosen as the variables to be analyzed, including CaCO_3_, Na_2_SO_4_, K_2_HPO_4_·3H_2_O, MnSO_4_·H_2_O, MgSO_4_·7H_2_O, FeSO_4_·7H_2_O, CuSO_4_·5H_2_O, and ZnSO_4_·7H_2_O, which were designated as X_1_, X_2_, X_3_, X_4_, X_5_, X_6_, X_7_, and X_8_, respectively. Each variable was evaluated at a high level (+) and a low level (−). The experimental design was developed by Minitab 16.0 (Minitab Inc., State College, PA, USA), as shown in Table 1. All the variables were evaluated in twelve experimental trials and the average Nsh yield for each trial was used as the response variable. The variables that were significant at 95% confidence level (*p* < 0.05) from the regression analysis were considered to have a significant effect on Nsh production and then further evaluated in optimization experiments. 

### 3.4. Central Composite Design

A spherical central composite design with three independent variables (Na_2_SO_4_, MnSO_4_·H_2_O, and MgSO_4_·7H_2_O) and five levels was used to determine the response pattern. Design Expert 8.0.7.1 (Stat-Ease Inc.) was used to generate experimental designs, estimate the responses of dependent variables, and generate the contour and response surface plots. The complete design included 20 experiments with six replications at the center point (Table 3). All experiments were performed in triplicate, and the average Nsh production was used as the dependent variable. Experimental data were fitted to a quadratic polynomial model, and the regression coefficients were obtained. 

The generalized response surface model is given below:(2)
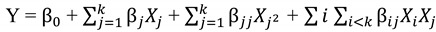

where *Y* is the estimated response, β_0_, β*_j_*, β*_jj_* and β*_ij_* are the regression coefficients for intercept, linearity, square, and interaction, *X_i_* and *X_j_* are the independent coded variables, and *k* is the number of variables, respectively.

The coefficient of determination (*R^2^*) and adjusted *R^2^* (Adj*R^2^*) were used to determine the significance of the second-order polynomial model [20]. The final model was displayed as three-dimensional (3D) response surface plots by varying two factor levels while keeping the third one at “0” level, and the optimal levels of factors could be calculated by first-order partial derivative from Equation (1).

### 3.5. Scale-up Fermentation Process

Scale-up fermentation was carried out in a 100 L fermentor (Bioengineering AG, Wald, Switzerland) with a 60 L working volume of statistically (RSM) optimized medium (40 g/L glucose, 40 g/L starch, 30 g/L soy meal, 2 g/L (NH_4_)_2_SO_4_, 2.5 g/L NaCl, 2.63 g/L Na_2_SO_4_, 0.21 g/L MnSO_4_·H_2_O, 3.37 g/L MgSO_4_·7H_2_O, 7.5 g/L CaCO_3_, 0.5 g/L K_2_HPO_4_·3H_2_O, 0.01 g/L FeSO_4_·7H_2_O, 0.01g/L CuSO_4_·5H_2_O, and 0.03 g/L ZnSO_4_·7H_2_O). The fermentor was an agitated bioreactor equipped with two turbine impellers and devices for on-line monitoring of pH, stirrer speed, pressure, and dissolved oxygen (DO). Inoculum containing the initial biomass was prepared in a set of holding tanks and then transferred into the fermentor by 10% inoculation. During fermentation, the pressure in the fermentor was maintained at 0.05 MPa by adjusting the escaping airflow. The temperature of the culture medium was 28 °C, and pH was controlled in the range of 6.8–7.2 by addition of 1 M H_3_PO_4_. The DO concentration was maintained above 30% of air saturation by adjusting the agitation speed and aeration rate during fermentation. A sterilized solution of 40% glucose and 2.5% (NH_4_)_2_SO_4_ (w/v) in water was fed at an interval of 72 h (400 mL), 120 h (200 mL) or 168 h (200 mL). Samples were taken every 8 h for analysis of Nsh production and biomass (%, wet cell weight per 100 mL fermentation broth by centrifugation at 1000 rpm for 10 min).

### 3.6. Analysis of Nsh Concentration

**Figure 4:**
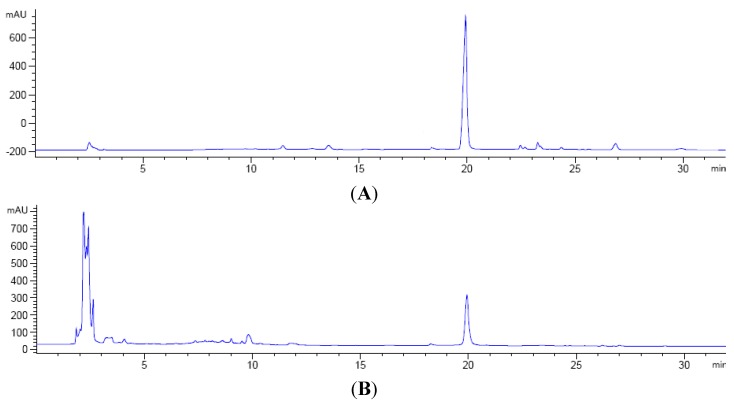
RP-HPLC chromatograms of Nsh standards (**A**) and fermentation samples of 208 h (**B**).

Samples were taken at different time points and extracted by adding the same volume of ethanol. A standard stock solution of Nsh was dissolved in *N*,*N*-dimethylformamide (DMF) at 1 mg/mL and diluted with appropriate volumes of DMF to prepare different working solutions. Quantitative analysis of fermentation samples was performed on an Agilent 1260 HPLC apparatus (Palo Alto, CA, USA) equipped with a quaternary gradient pump. An Eclipse C18 analytical column (4.6 mm × 250 mm, 5 μm, Agilent Technologies) was used at a column temperature of 40 °C, with a flow rate of 1.0 mL/min and sample injection volume of 20 μL. The eluents were monitored with a diode array detector at 254 nm. The mobile phase consisted of solvent A (H_2_O, 0.1% TFA) and B (CH_3_CN, 0.1% TFA) with the following profile: 0–3 min, 15% A and 85% B; 3–6 min, 15% → 40% A and 85% → 60% B; 6–12 min, 40% A and 60% B; 12–19 min, 40% → 55% A and 60% → 45% B; 19–22 min, 55% → 85% A and 45% → 15% B; 22–28 min, 85% A and 15% B; 28–32 min, 85% → 15% A and 15% → 85% B. HPLC data were acquired using the Lab Solutions software (Agilent Technologies Inc., Waldbronn, Germany) (Figure 4).

### 3.7. Data Analysis

Data were expressed as means ± standard errors of the means (SEM) of triplicate experiments. Statistical analysis of the experimental data was carried out by Minitab 16.0 (Minitab Inc.) and Design Expert 8.0.7.1 (Stat-Ease, Inc.). ANOVA was used to estimate the statistical parameters, and the fitness of the polynomial model equation was expressed by the coefficients of *F*-test and *p*-value. The significance level for all comparisons was set at *p <* 0.05.

## 4. Conclusions

Nsh is a very promising thiopeptide antibiotic against antibiotic resistant bacteria, and there is a growing research interest in the biosynthesis of Nsh. This study shows the advantage of using RSM over traditional techniques to investigate the effect of mineral salts on Nsh fermentation. Na_2_SO_4_, MnSO_4_·H_2_O, and MgSO_4_·7H_2_O were proved to have significant effects on Nsh production and a regression equation model was obtained to determine the interactive effect of these salts on the production of Nsh. Validation experiments were also carried out in scale up fermentation to verify the accuracy of the model and a maximum production of 1501 mg/L was achieved under optimal mineral salts combination, which closely matched the predicted yield and resulted in 1.56 times higher in terms of original productivity. Thus, this research proves the reliability of RSM in Nsh fermentation optimization and can serve as a fundamental study to further evaluate the potential of other conditions.
